# Role of High-dose Chemotherapy and Autologous Stem Cell Transplantation for Relapsed Ewing's Sarcoma: A Case Report with Focused Review of Literature

**DOI:** 10.7759/cureus.2581

**Published:** 2018-05-05

**Authors:** Pavan Tenneti, Umar Zahid, FNU Sagar, Muhammed Usman, Faiz Anwer

**Affiliations:** 1 Department of Medicine, Banner University Medical Center Tucson, Tucson, USA; 2 Internal Medicine, University of Arizona, Tucson, USA; 3 Hematology and Oncology, University of Arizona, Tucson, USA

**Keywords:** relapsed, ewing's sarcoma, stem cell transplantation, high-dose chemotherapy

## Abstract

We report a case of a patient with relapsed Ewing’s sarcoma (ES). After receiving conventional chemotherapy (CC) and noticing chemosensitivity of the disease, we proceeded to give the patient two separate cycles of HDCT consisting of a melphalan/busulfan regimen in the first cycle and etoposide/melphalan in the second cycle. The patient proceeded to get an autologous stem cell transplant (ASCT) after each cycle of HDCT. Our patient, despite multiple poor prognostic factors, including advanced age and multiple sites of disease relapse, showed a one-year event-free survival.

Relapsed ES is associated with a poor prognosis. No treatment regimen has yet been established as a standard of care in patients with relapsed ES. We conducted a focused literature review to assess the efficacy of high-dose chemotherapy (HDCT) followed by ASCT in patients with relapsed ES. Given the improved survival outcome with HDCT followed by ASCT in our patient, we propose that its role in relapsed ES needs further assessment through large prospective, randomized controlled studies.

## Introduction

Patients with localized primary Ewing’s sarcoma (ES) have a five-year overall survival (OS) of 60 - 70% with the use of multimodality treatment [1]. In patients with primary metastatic ES, the five year OS rate is 20 - 40% with treatment [1]. Approximately 30 - 40% of patients with localized primary ES who initially achieved remission after front-line treatment experience disease relapse, and the prognosis in these patient groups was shown to be dismal with one and five year OS of 43% and 13%, respectively [2]. At the time of disease relapse, prognostic factors indicative of poor outcome include relapse time less than two years from initial diagnosis, the location of relapse at the extrapulmonary site, combined local as well as systemic relapse, and abnormally high lactate dehydrogenase (LDH) levels at initial diagnosis [3-5]. No standardized treatment has been approved for the treatment of relapsed ES. Local therapy at the site of relapse, including radical surgery, has been shown to be beneficial [5]. Conventional chemotherapy (CC) regimens given at relapse have led to response rates up to 29 - 68.1%; response depended on the type of regimen used and site of relapse [6-10]. The event-free survival (EFS) at one to two years has been noted to be between 22.7 - 26% in a couple of studies [8-9]. OS rates at one to two years in other studies were shown to be about 28 - 61% [7-8]. The five year OS was 20 - 24.5% in another retrospective study [11]. 

Despite its reported survival benefit as a consolidation treatment after CC, high-dose chemotherapy (HDCT) and autologous stem cell transplant (ASCT) are not routinely used in the United States for relapsed Ewing’s sarcoma. We present a focused literature review, along with a case report of a patient diagnosed with chemosensitive relapsed ES with an expected poor long-term prognosis based on his poor prognostic markers at relapse, who received two cycles of HDCT followed by ASCT.

## Case presentation

**Figure 1:**
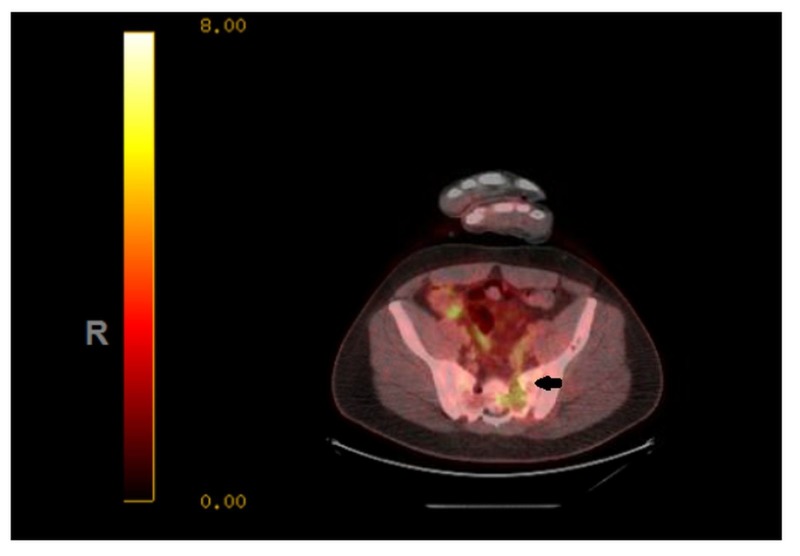
MRI scan of the pelvis (2012) - Ewing's sarcoma in left sacrum/ilium locally involving neuroforamina, paraspinous, and gluteal muscles MRI: magnetic resonance imaging

**Figure 2:**
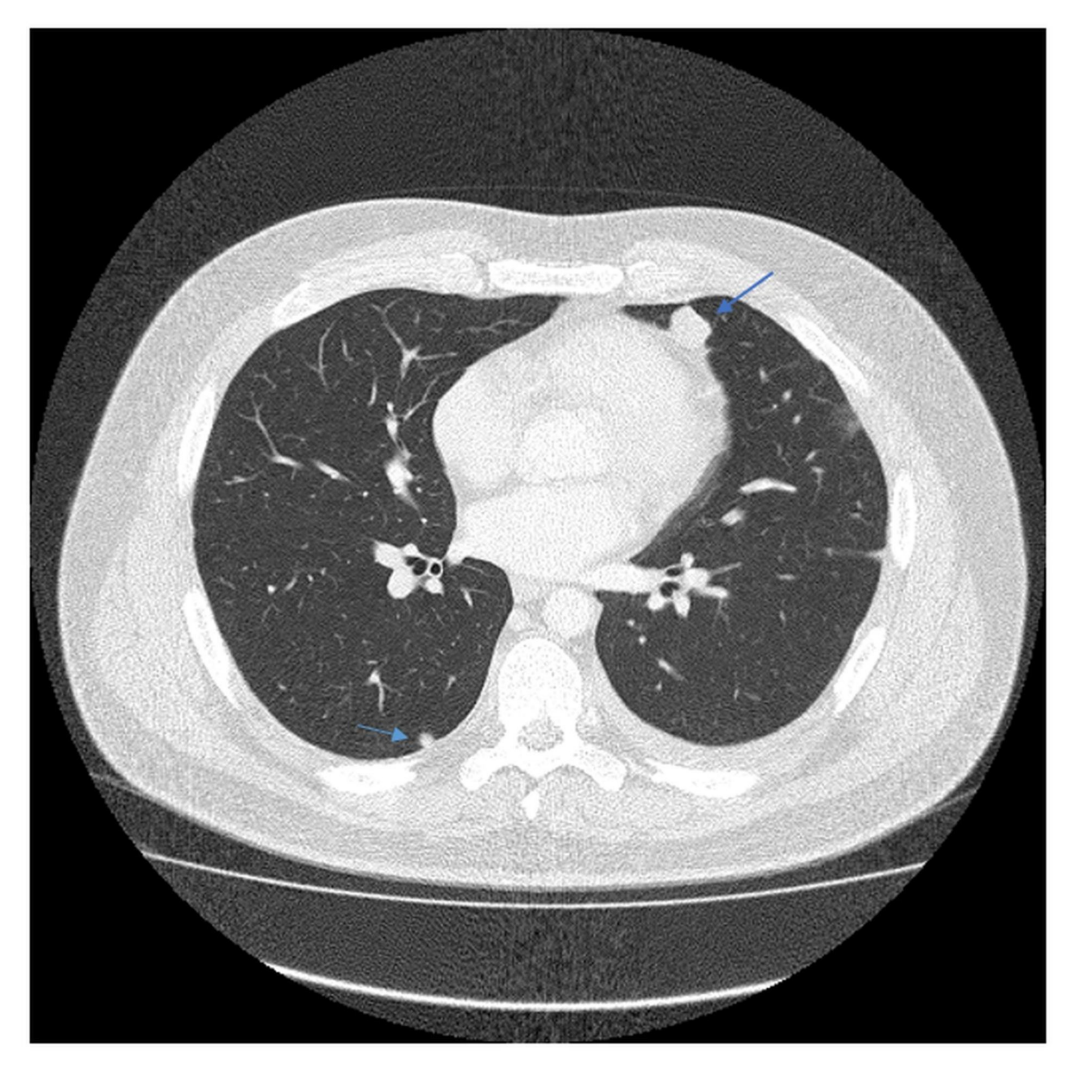
CT scan chest (May 2014) Relapsed Ewing's sarcoma in the lungs with multiple pulmonary nodules, the largest being 11.8 x 9.8 mm (arrow on right) CT: computed tomography

**Figure 3:**
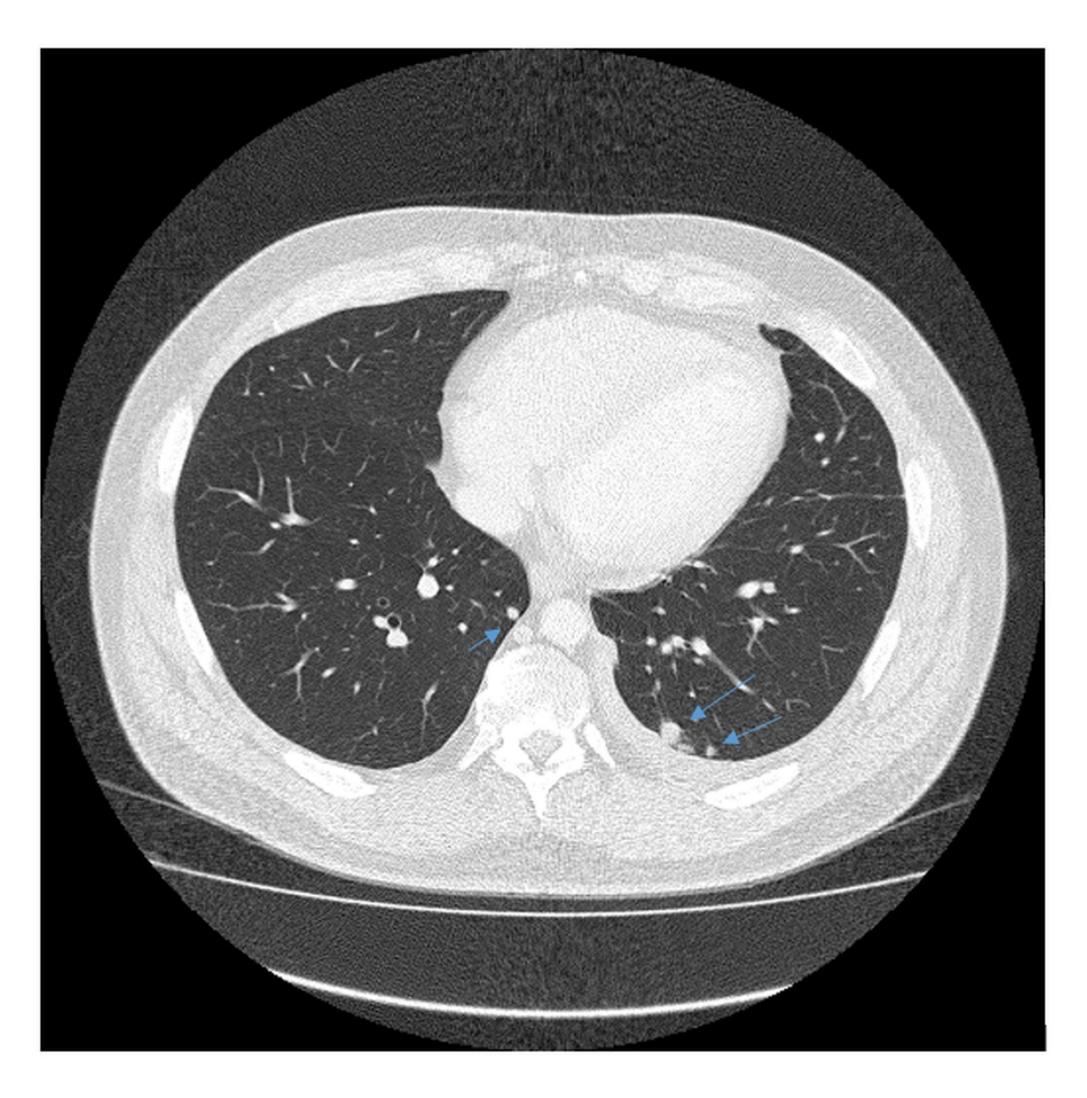
CT scan chest (May 2014) Relapsed Ewing's sarcoma in the lungs with multiple pulmonary nodules CT: Computed tomography

A 35-year-old Caucasian male presented during February 2012 with a three-month history of progressive lower back pain radiating to the left leg. Dorsal spine magnetic resonance imaging (MRI) revealed a mass involving the left ilium, sacrum, and left sacroiliac joint. It was also invading the S1-S2 left neural foramen and superior sciatic notch (Figure 1). Biopsy of the mass showed a small round blue cell malignant neoplasm, having a uniform site of morphology with a lobulated growth pattern with some of the cells having limited amounts of amphophilic cytoplasm. There was a strong immune positivity for CD99 but negative for desmin CD 163 and CD68. He was diagnosed with primary localized ES. The patient received neoadjuvant chemotherapy and adjuvant radiation therapy according to the VIVA (vincristine + ifosfamide + doxorubicin + actinomycin D) regimen. He completed radiotherapy to the primary site in August 2012 with concurrent ifosfamide and etoposide. All planned treatment was completed in January 2013. The patient was under close follow-up, and in May of 2014, he presented with multiple lung and two pleural lesions. Biopsy confirmed the lesions to be a relapse of ES with metastasis to lungs (Figures 2, 3). In addition, pleural fluid immunohistochemical stains demonstrated the neoplastic cells to be positive for CD99 and negative for MAK-6, synaptophysin, neuron-specific enolase (NSE), and CD56, consistent with metastatic ES. The patient received five cycles of topotecan and cyclophosphamide. A follow-up computed tomography (CT) of the chest in July 2014, before cycle 3, showed interval decrease in the metastatic lesions, consistent with chemosensitive disease. A positron emission tomography/computed tomography (PET/CT) scan during August 2014, after five cycles of topotecan/cyclophosphamide, showed stable metastatic disease in the form of pulmonary nodules and pleural involvement. Autologous stem cells were collected during a single leukapheresis session before the first high-dose chemotherapy (HDCT). The patient received high-dose chemotherapy in October of 2014 (busulfan, 0.8 mg/kg IV (intravenous) q six hours x 16 doses; melphalan, 140 mg/m^2^) and received CD34+ cells 4.24 x 10 e6/kg infused as an autologous stem cell rescue. The second round of planned consolidative high-dose chemotherapy was given during July 2015 (etoposide/melphalan regimen - etoposide, IV 400 mg/m^2^, total three doses (Days 2, 3, 4) and melphalan, IV 100 mg/m^2^, one dose (on Day 1)) followed by autologous peripheral blood stem cell rescue. During the first cycle of chemotherapy, the patient developed mucositis, neutropenic fever, and diarrhea secondary to Clostridium difficile colitis. After the second cycle of HDCT, the patient developed mucositis and transient elevation of liver function tests but these abnormalities resolved over a few weeks.

**Figure 4:**
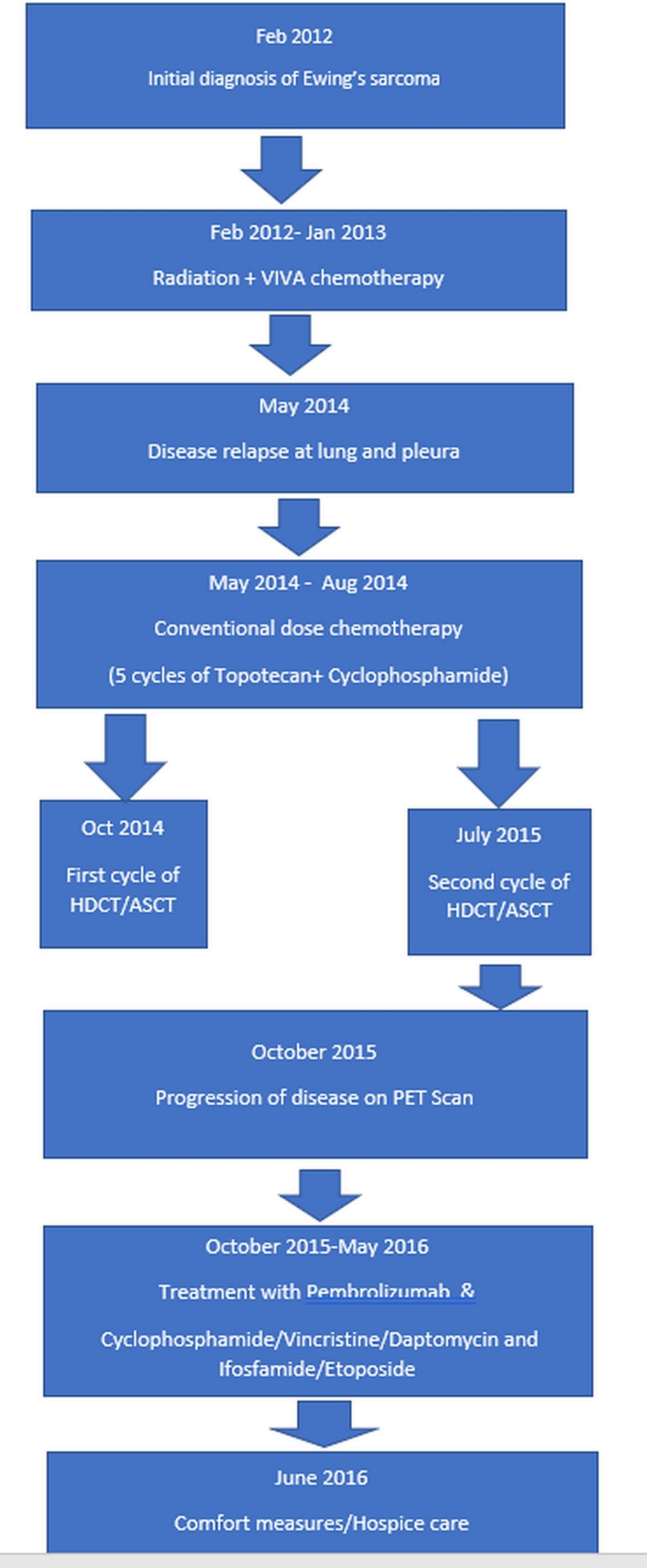
Flowchart showing sequence of events in patient history ASCT: autologous stem cell transplant; HDCT: high-dose chemotherapy; PET: positron emission tomography; VIVA: vincristine + ifosfamide + doxorubicin + actinomycin D

HDCT achieved 12 months of progression-free survival (PFS); however, on a subsequent PET scan performed during October 2015, the patient showed disease progression. An increase in the size of the metastatic lesions was noted, along with new areas of involvement of malignancy. The patient subsequently received six cycles of pembrolizumab, an antibody against programmed cell death 1 (PD-1) receptor. Subsequent imaging studies showed a progression of the disease, and the patient subsequently received cyclophosphamide, vincristine, dactinomycin (alternating with ifosfamide), and etoposide during March of 2016. The patient developed significant toxicities, bone marrow suppression, and in the presence of progressive disease during June 2016, he chose hospice care (Figure 4).

## Discussion

In patients with relapsed ES, there is no established standard of care salvage chemotherapy regimen. Regarding conventional salvage options, different regimens have been tried with variable results (Table 1). A phase II study using the ICE (ifosfamide, carboplatin, and etoposide) regimen showed an overall response rate (ORR) of 51% and one-year (yr) and two-yr OS rates of 49% and 28%, respectively [7]. Studies conducted with non-ifosfamide regimens, such as docetaxel along with gemcitabine, showed an ORR of 29% and a median duration of response of 4.8 months [6]. The ORR was 44%, with a two-yr EFS of 26% in a study with 54 relapsed ES patients when cyclophosphamide and topotecan were used [8]. Irinotecan and temozolomide resulted in an ORR of 63% and a time to progression of eight months [10]. The ORR was 68% with an EFS of 22.7% at 10.3 months in a study where patients received the VIT (vincristine, irinotecan, temozolomide) regimen [9]. Another retrospective study on relapsed ES, conducted on 107 patients using either etoposide and cisplatin or etoposide and carboplatin, showed a median EFS of 6.5 and 14 months, respectively, with five-yr OS rates of 20% and 24.5%, respectively [11]. Collectively, these data suggest the need for further improvement in the treatment outcomes in ES patients.

**Table 1:** Outcomes of Relapsed Patients in Studies Utilizing Conventional Dose Chemotherapy Ca: carboplatin; Ci: cisplatin; Cy: cyclophosphamide; D: docetaxel; E: etoposide; G: gemcitabine; I: ifosfamide; Ir: irinotecan; m: months; Te: temozolomide; To: topotecan; V: vincristine; NL: Not listed

Study	Agents used	Number of patients	Response rate	Overall survival	Event free survival
Navid et al. [6]	G + D	22	29%	NL	NL
Van Winkle et al. [7]	I + Ca + E	97	51%	1 yr: 49% 2 yr: 28%	NL
Hunold et al. [8]	To + Cy	54	44.4%	1 yr: 61%	23.1 m = 25.9%
Raciborska et al. [9]	V + Ir + Te	22	68.1%	NL	10.3 m = 22.7%
Casey et al. [10]	Ir + Te	20	63.1%	NL	NL
Van Maldegem et al. [11]	E + Ca/Ci	107	NL	5 yr: 20 - 24.5%	NL

Patients presenting with distant site, non-pulmonary metastasis at the time of relapse have particularly poor long-term outcomes with the use of conventional chemotherapy regimens. In a 2009 retrospective study by Casey et al. with 20 patients with refractory/relapsed ES who were treated with irinotecan and temozolomide, the median time to progress in patients with relapse at multiple sites (n = 6) was 2.4 months in comparison to 24.3 months (p = 0.00) for patients with single site disease relapse (n = 9) [10]. In another retrospective study by Leavey et al. in 2008 (n = 262), the median duration of survival for patients with distant relapse, other than lung, was nine months [4]. Bacci et al., who published a study of 195 patients in 2003, noted the EFS at five years for a subgroup of patients with distant relapse was 8.6%. The median duration of survival was 19.3 months [3]. In another study of 71 patients, Rodriguez-Galindo et al. showed a five-year OS for patients with a distant relapse to be only 17.5% [5].

HDCT, followed by ASCT, is based on the observation that the dose intensity of chemotherapy can determine outcomes in many malignancies. Dose-response is steep for both toxic and therapeutic effects. Preclinical studies documented a direct correlation between the dose of chemotherapy and tumor cytotoxicity. A multiple log increase in tumor cell death can be possible with a three to tenfold increase in the dosage of chemotherapy, particularly for alkylating agents. Myelosuppression is one of the major side effects at this dose [12]. ASCT helps to counter this by rescuing the marrow and allows for dose escalation [13].

Through a systemic literature search, we identified seven retrospective studies where HDCT, followed by ASCT, was given in patients with relapsed ES (n = 708) (Table 2). The outcomes of patients with local and metastatic relapse who received HDCT followed by ASCT after CC were studied by McTiernan et al. 2006 (n = 33) [14]. The two and five-year EFS rates were 43 and 39%, respectively. The corresponding OS rates were 51 and 43%, respectively. Fifty-five patients with local and metastatic relapse were studied by Barker et al. in 2005 [15]. All patients were treated with CC; in addition, HDCT, followed by ASCT, was given to patients who showed either complete response(CR) or partial response (PR). The EFS and OS rates at five years for patients treated with HDCT/ASCT was 61 and 77%, respectively. On the other hand, the reported five year EFS and OS for patients that were treated with only CC was 7% each (p values not reported). In an additional study by Ferrari et al. in 2015, the five-year OS rate was 50% for patients who received CC followed by HDCT/ASCT (n = 107) [16]. Shanker et al. 2003 [17] reported OS of 28% at 3.5 years for patients with relapsed ES who got HDCT/ASCT after CC. The OS of 21% at three years was reported by Palmerini et al. (Palmerini et al.: High-dose chemotherapy with autologous stem cell transplantation for relapsed Ewing's sarcoma-abstract, ASCO annual meeting, May 2009. http://ascopubs.org/doi/abs/10.1200/jco.2009.27.15s.10545) in another study for patients with relapsed ES where HDCT/ASCT was given after CC. The outcomes of patients from the CESS (Cooperative Ewing Sarcoma study group) registry with local and metastatic relapse was reported by Rasper et al. 2014 (n = 239) [18]. All patients received CC. In addition, 73 patients who showed chemosensitivity (either CR/PR) received HDCT/ASCT. The two- and five-year EFS for patients who were treated with HDCT were 44 and 24%, respectively, whereas they were 10% and 6%, respectively, in patients treated with only CC (p = 0.01). The two- and five-year OS rates for patients who received HDCT were 66% and 42%, respectively, compared to 22% and 10% in patients with only CC (p = 0.01). The outcomes of 138 patients with metastatic disease at relapse was studied by Bacci et al. [3]. Ninety-five patients who received only CC did not survive longer than five years. Also, none of the 15 patients who received HDCT as the induction treatment without CC survived longer than five years, suggesting that CC may be required, followed by HDCT consolidation to further improve survival. To summarize, for most studies consisting of only relapsed patients (except for one), EFS rates at two and five years ranged from 42 - 44% and 24 - 43%, respectively. The OS rates at two and three to five years ranged from 50 - 66% and 21 - 50%, respectively. The patients in the Bacci et al. study did not do well because the disease was metastatic at relapse and they did not get CC prior to HDCT [3].

**Table 2:** Studies Using HDCT and ASCT in Relapsed Ewing's Sarcoma A: dactinomycin; ASCT: autologous stem cell transplant; C: cyclophosphamide; Ca: Carboplatin; CR: complete response; D: doxorubicin; E: etoposide; EFS: event-free survival; HDCT: high-dose chemotherapy; I: ifosmade; Ir: irinotecan; M: methotrexate; NL: Not listed; No: number; OS: overall survival; PR: partial response; SCT: stem cell transplant; T: topotecan; TBI: total body irradiation; Te: temozolomide; V: vincristine; Ω: topotecan, Cytoxan, irinotecan; C1: High-dose chemotherapy regimen 1; C2: High-dose chemotherapy regimen 2; PSBC: Peripheral blood stem cells: BSC: Bone marrow stem cells.

Study	Median age (yr.)	No. of pts	Type of relapse (No. of pts)	Chemotherapy	HDCT (mg/m^2^; mg/kg)	Source of stem cell	Response (No. of pts)	EFS	OS
Rasper et al. 2014 [18]	NL	239	Local (42), Distant (142), Combined (30)	T/C, I/Te, If/E/Carboplatin	C1: Busulfan/Melphalan (NL); C2: Treosulfan/Melphalan (NL); Others: Ω	NL	C1: CR (5) PR (2) C2: CR (19) PR (8)	2 yr HDCT – 44% w/o HDCT-10% (p value: 0.01); 5 yr. HDCT – 24% w/o HDCT - 6% (p value: 0.01)	2 yr HDCT-66% w/o HDCT-22%; 5 yr HDCT - 42% w/o HDCT - 10% (p value: 0.01)
Barker et al. 2005 [15]	13.5	55	Local (6); Distant (39); Combined (10)	VDC - IE; VACIME; VDC-A; VDC-A-M	Busulfan/melphalan/ thiotepa (NL)	PBSC	CR/PR (27)	5 yr HDCT: 61% w/o HDCT: 7%	5 yr HDCT: 77% w/o HDCT: 7%
Ferrari et al. 2015 [16]	NL	107	Local (11); Distant (96)	VDCAIE	Busulfan/melphalan (4 mg/kg and 140 mg/m^2^)	PBSC	NL	NL	HDCT: 5 yr OS 50%
Palmerini et al. 2009 abstract	17	72	Local (11)	NL	Busulfan/melphalan (4 mg/kg and 140 mg/m^2^) Palliative	NL	NL	NL	HDCT: 3 yr OS 21%
McTiernan et al. 2006 [14]	19	33	Local (11) Distant (18); Combined (4)	Ca, E C A I D V	Busulfan/melphalan (600 mg/m^2^/140 mg/m^2^) - 22 pts Melphalan/etoposide (130 mg/m^2^/60 mg/m^2^) - 7 pts TBI Melphalan/Cytoxan (16 mg/kg/60 mg/kg) - 1 pt. Melphalan (150 mg/m^2^) – 3 pts	PBSC BSC	Prior to HDCT: CR (14) PR (10) Post-HDCT: CR (21) PR (2)	2 yr EFS 42.5%; 5 yr EFS 38.5%	2 yr OS 50.7%; 5 yr OS 42.8%
Shankar et al. 2003 [17]	14	64	Local (11)	Ca/E, C/E /Ca, C/E	Melphalan (NL) TBI	PBSC BSC	NL	3.5 yr EFS 14%	3.5 yr OS 28%
Bacci et al. 2003 [3]	18	138	Distant (138)	none	Melphalan (NL) Busulfan (NL)	PSBC BSC	NL	NL	5 yr only HDCT – 0% w/o HDCT – 0%

The patient reported in our case report was an adult male with relapsed ES who had a relapse at multiple sites, including the lung and pleura. Based on the review of poor prognostic factors, patients with non-pulmonary relapse or relapse at multiple distant sites have a poor prognosis with traditional chemotherapy. In addition, some studies showed that patients with advanced age have poor outcomes with ES [19]. We decided to give HDCT with ASCT after CC in a patient who had chemosensitive relapsed ES, as our literature review showed a possible benefit of HDCT for such patients. Our patient received two cycles of HDCT followed by ASCT, achieved about 12 months of PFS, and progressed four months after completion of the second cycle. Subsequently, it did not show a response to other treatments, including PD 1 inhibitor (pembrolizumab) and additional salvage chemotherapy. He ultimately chose hospice care.

## Conclusions

Patients with relapsed ES are associated with a poor prognosis when treated with conventional chemotherapy. The worst prognosis is seen in patients with multiple distant and non-pulmonary sites of relapse. Through a literature search, we identified studies that showed the potential benefit of HDCT followed by ASCT in chemosensitive disease in relapsed ES. Given the positive results in the literature (mainly, multiple large retrospective studies), the role of HDCT followed by ASCT in relapsed ES needs to be further defined by prospective, randomized controlled studies.
